# Scientific Proceedings of the Richmond Academy of Medicine and Surgery

**Published:** 1890-10

**Authors:** J. W. Henson

**Affiliations:** Reporter


### SCIENTIFIC PROCEEDINGS OF THE RICHMOND ACADEMY OF MEDICINE AND SURGERY.

---

J. W. HENSON, REPORTER.

---

AUGUST 26th, 1890.

Dr. W. W. Parker, President in the Chair.

Symptoms of cocaine poisoning from one-half drachm (per rectum) of a three per cent. solution.

Dr. Ramon L. Garcin reported that, six weeks or two months since, he was called to see Mr. F——, who had been operated upon by an irregular, who was out of the city when Dr. Garcin was called. When the latter reached the man he was suffering intensely, and morphia hypodermically not relieving him, one-half drachm of a three per cent. solution of cocaine was administered per rectum. No relief of pain ensued, but in a short time breathing became quickened, extremities cold, pulse rapid and weak. The man described his flesh as tingling like the sensation felt upon first grasping the poles of a galvanic battery.

By the use of stimulants he soon rallied.

Dr. J. P. Roy asked if the muscles of deglutition were affected.

Dr. Garcin.—No.

A CASE FOR DIAGNOSIS.

On July 8th, 1890, Dr. Garcin said he was called to see Mr. M——, aged nineteen years, who complained of intense nausea and pains, resembling cramps, in the region of the epigastrium. The history of the case before this was negative.

He had been to work up to the day before taking his bed, although a week previously there was a slight diarrhœa for a few days, but when the doctor was called the man said his bowels were in a normal condition.

His tongue was very slightly furred, temperature (by mouth) 98½° F.; the abdomen, especially about the umbilical region, was very tympanitic. A distinct gurgling (exactly resembling that of typhoid fever) was present in both iliac fossæ; the bowels had been moved once that day.

The doctor gave simple anodyne for the cramp (which soon afforded relief), and dilute hydrochloric acid—fifteen drops every four hours.

July 9th and 10th.—Patient about the same, bowels acting once daily. The doctor ordered a mixture of equal parts of turpentine and sweet oil over the iliac region. He from the first ordered liquid diet. The temperature was normal, taken once that day.

July 12th.—The characteristic diarrhœa of typhoid, "pea soup" discharges; temperature normal, morning and evening. Tympanites being more decided, fifteen drops of turpentine, in emulsion every six hours, were ordered.

July 13th, 14th, 15th.—Tympanites decidedly diminished. Diarrhœa worse towards evening, three, four and sometimes five discharges from 3 P. M. to 6 or 7 P. M. The doctor ordered 15 gtt. dilute hydrochloric acid, 10 grs. each of lactopeptine and bismuth subnitrate every four hours.

July 16th and 17th.—Patient seen by Dr. R. T. Ellis for Dr. G.

July 18th, 19th, 20th and 21st.—Bowels not so bad, tympanites had disappeared, slight gurgling in right iliac fossa—no fever.

July 22d.—Pulse and temperature normal, bowels moved once, tongue healthy.

The interesting features of this case, said the doctor, were: (1) Entire absence of fever (morning and evening). (2) Absence of typhoid tongue. (3) Absence of coma, the man being conscious throughout the attack.

The after treatment was a tonic of vin mariani. Patient was out by August 1st.

Dr. Parker.—Any history of phthisis?

Dr. Garcin.—No.

Dr. J. M. Winfree.—Any pain, mucus or blood?

Dr. Garcin.—Some pain; no mucus or blood.

Dr. T. J. Moore.—How long was the man sick?

Dr. Garcin.—About three weeks.

Dr. Moore.—Did Dr. G. see him when first taken?

Dr. Garcin.—Yes, when he first took to bed, but had been complaining before, although at work up to the day before the doctor's first visit.

Dr. Moore.—What was his work?

Dr. Garcin.—Apprenticed lithographer.

Dr. Moore.—Did he work in lead?

Dr. Garcin.—Yes, a little in mixing colors.

Dr. Moore stated that the symptoms were so obscure it was impossible to make anything like an accurate diagnosis.

When a person was subject to the gradual and prolonged absorption of lead, there occurred sometimes a condition where there was no manifestation of colica pictonum proper, but a certain degree of constipation, followed by an irritative diarrhœa. *Possibly* this patient was so affected. The diurnal normal temperature excluded typhoid fever.

We sometimes have a condition of bowel where a local irritation of a diarrhœic character congests and causes ulceration of

Peyer's patches; this might give you the characteristic pultaceous stools, with the fetor of typhoid actions accompanied by tympanites. Mere tympanites occurred in so many conditions that it was not calculated to lead up to a diagnosis. Tenderness in the ilio-cæcal region was more directly prognostic.

### ABSCESS OF THE PAROTIDS COMPLICATING TYPHOID FEVER.

Dr. Parker had seen in a boy, aged sixteen years, abscess of each parotid gland as a complication of typhoid fever. Each discharged from the ear before being lanced, the discharge through the ear ceasing after the lancing. The point was that the boy recovered, though some one had stated that all cases of typhoid fever with abscess about the parotid gland proved fatal.

### FATALITY IN SUPPURATIVE PAROTITIS COMPLICATING TYPHOID FEVER.

Dr. Moore said that several years since Dr. R. M. C. Page, of New York, wrote an article on secondary parotitis, in which he stated that when suppuration of the parotid gland is seen as a complication of typhoid fever, nearly all cases so affected proved fatal.

Dr. Roy had a similar case to Dr. Parker's last fall, occurring about the third week of typhoid. As in Dr. Parker's case, both abscesses discharged from the ear. There was also an accompanying cancrum oris, a spot of gangrene the size of a silver dollar appearing on outside of the cheek before death, which followed soon.

### ANEURISM OF THE ARCH OF THE AORTA.

Dr. Lewis C. Basher was called in consultation with Dr. Jones to see a colored woman who was suffering from the effects of a pulsating tumor occupying the upper part of the left side of the throat. He found a patient about thirty-five years of age, who was exceedingly emaciated and suffering greatly from pain, dyspnœa and extreme debility. She had little or no appetite. On examination, the tumor, which measured about 3x3½ inches at every point, gave a distinct pulsation, corresponding to the cardiac systole. The stethoscope gave only an indistinct "bruit."

The sternum appeared to project forward, and there was a complete dislocation of the left clavicle at the left sterno-clavicular articulation. He (Dr. Basher) diagnosed the tumor as an aneurism of the arch of the aorta, which had projected forward, pressing against the sternum ribs and clavicle, and causing absorption of the former (sternum and ribs), and dislocation of the latter (clavicle).

On Saturday night last this patient died, and yesterday, with the assistance of Drs. C. A. Blanton, David J. Coleman, Jones, and others a *post mortem* was made, which confirmed the diagnosis. A sacculated aneurism springing from the arch of the aorta, and projecting forward and upward, had dislocated the left clavicle at its sternal end, and had caused absorption of some of the upper ribs, as well as the sternum at the junction of the manubrium and gladiolus. There was a slight rupture in the sac, from which there was probably a slow leakage of blood, thus accounting for the gradual, rather than the sudden death, such as results from sudden rupture and copious hemorrhage. The left side of the thorax was filled with blood. The doctor then exhibited the aneurism to the meeting.

### SHOULD SET A LIMIT TO LIFE IN ORGANIC HEART DISEASE, WITH CAUTION.

Dr. Parker had reported two or three years ago the case of a man, aged about seventy-five, with enlarged and valvular disease of the heart. Pulse was twenty-four per minute for months at a time. While under Dr. Parker's observation, a period of two months, he used to apparently die four or five times a day. At the end of said two months he left town--now over two years ago. He just died a few days ago.

A doctor should be careful how to limit life in a person with organic heart trouble. As illustrative, the doctor told of a man named Shook, the action of whose heart (from hypertrophy) was so violent as to shake the bed. After being in bed for several months he got up and walked about for one or two years.

### MASTOIDITIS IN THE NEGRO.

Dr. W. F. Mercer asked if anybody had ever seen mastoiditis in a full-blooded negro. Dr. T. E. Murrell, of Little Rock, Ark., had stated that mastoiditis was never seen in a full-blooded negro. He (Dr. Mercer) in a dispensary practice of six years, (the majority of the patients, negroes too), had never seen a case in a full-blooded negro until within the last two months. One case, a man. His only evidence that he was full-blooded was his appearance and statement. Dr. Parker had a case in a mulatto. This was operated upon by Dr. J. A. White, but death occurred three or four weeks afterwards.

### CONTINUED FEVERS.

Dr. Parker saw some time ago a case of fever with Dr. O. A. Crenshaw, who is a great believer in typhoid fever,--insisted that this was typhoid for sometime, but it was not. The woman had been badly treated by her husband. Dr. Parker thought it an irritative fever. It terminated favorably after three or four weeks' duration. It was not usual, though, to see a continued fever unless it be typhoid. He (Dr. Parker) had seen numbers of cases of slow pulse typhoid before the war. The amount of prostration, however, proved them to be typhoid to his mind. He was now attending a young lady who had had typhoid fever in Charlottesville. Supposed to be decidedly convalescent, she was brought here two weeks ago to escape diphtheria. Moving did a great deal of harm. Her temperature was now 103° F. She could not walk five steps now without help. He was called Saturday night to see a remarkably handsome young man in the same house with the young lady.. His temperature was 103°, and he presented the symptoms of cold flushes, and steams alternating with cold chills. The doctor (Dr. Parker) thought it a general inflammatory fever or sort of general rheumatism. He gave him calomel and soda then, and on Sunday quinine, five grains every four hours. Monday he was in a profuse cold sweat, pulse feeble, no fever from symptoms (the doctor having left his thermometer behind), bowels and tongue pretty good, appetite bad except for liquids. The doctor thought he would soon be better. Tuesday (to-day) his temperature was 103°, perspiratfon gone, skin hot. Dr. Parker forgot to say he had two hemorrhages from the nose Monday night. He thought the case peculiar, and was uncertain whether it was typhoid or not.

Dr. M. D. Hoge, Jr.—Any cough?

Dr. Parker.—None.

Dr. David McCann.—Had Dr. Parker given him any antipyrine?

Dr. Parker.—None. Had Dr. Moore seen any case of continued fever not typhoid?

Dr. Moore.—Yes.

Dr. Parker.—What was the pathology of them?

Dr. Moore stated that every fever was continued in which there was no intermission.

Example.—Remittent malarial fever.

What was Dr. Parker's idea of a continued fever?

Dr. Parker thought typhoid and typhus continued fevers, but not malarial fever.

Dr. Moore stated that he had seen a form of fever this season that corresponded neither in type nor characteristic to either remittent or typhoid fever. He thought the lines were too sharply drawn by the writers concerning the continued fevers, common to various sections of the United States. We have occasionally in this part of Virginia a form of fever congestive in type (attributed to heat and the peculiar atmospheric conditions), with high and regular diurnal thermometric ranges, great rapidity of pulse, congestion and tenderness of spleen and liver, congestion of kidney, a certain amount of tympanites, and of uncertain duration, lasting often from ten days to two weeks. It did not yield to quinine, while alterative doses of mercury modified the disease, and shortened the duration of the fever. It was accompanied by a bilious diarrhœa, yielding best to bismuth and opium.

Typhoid existed in all parts of the United States. It is most prevalent and violent in type in high altitudes. It hugged the mountains and a belt of Piedmont country contiguous thereto. It was also found in the low country, and in tertiary formations. It is modified in many of its symptoms by the effect of prolonged heat, and the structural alterations of the glandular organs, particularly the spleen and liver by malarious influences.

The doctor was not referring to the disease called typho-malarial fever. The cause of typhoid fever has never been ascertained. Scientific men have offered many suggestions without definite results. Sewer gas was often mentioned as the vehicle by which the specific poison is conveyed. In many places, especially mountainous sections, both sewers and sewer gas were unknown. Running and well water were both frequently thought to contain the specific poison. He didn't believe a specific cause or germ had been definitely ascertained.

He related how an old Tennessee doctor, unlettered but experienced, at some convention, stated that he knew nothing of germs and the other "new fangled" notions in regard to the cause of typhoid fever, but that whenever he could induce any of his families to locate their stables and hog pens at a sufficient distance from their houses, and remove their chip piles at the proper season he noticed that such families were not troubled with typhoid fever.

Typhoid differed markedly in regard to the severity of attacks embracing from the walking cases upon the one hand to the malignant upon the other.

In regard to the perspiration mentioned by Dr. Parker, he had seen, frequently, profuse sweats in the first week of inception of typhoid fever, but usually there was a dry skin. Dr. Parker remarked that in some parts of East Tennessee the people forty years ago had never heard of sewers or sewer gas, *nor* of typhoid fever. But he spoke of an old gentleman owning about seventy-five negroes, and who lived seven miles from his nearest neighbor, and the fact that awhile later typhoid broke out among his slaves, though it had not been known of before.

